# Humanities Across Clerkships: A Longitudinal Course on Professional Identity Formation

**DOI:** 10.1007/s40670-025-02485-7

**Published:** 2025-08-23

**Authors:** Nabeel Akhtar, Rachel Casas

**Affiliations:** Division of General Internal Medicine, Department of Medicine, Penn State Health Milton S. Hershey Medical Center, 500 University Drive, Hershey, PA 17033 USA

**Keywords:** Curricular development, Undergraduate medical education, Medical humanities, Professional identity formation

## Abstract

Humanities Across Clerkships (HAC), a longitudinal course during third-year clerkships, is an innovative approach to promote reflection on professional identity formation and career development for medical students. This course was feasible and encouraged individual and group reflective practices, and it was positively received by medical students.

Professional identity formation (PIF) is a transformative process where an individual internalizes values and beliefs of a profession formed by personal and social experiences [1]. Medical students are expected to form a professional identity to choose and prepare for residency. In turn, medical educators have created innovative tools to promote PIF such as artwork, narrative writing, mentorship programs, and focus groups [1]. These interventions are often under Medical Humanities given the focus between self and the healthcare environment.

Medical students in third-year clerkships experience emotional and psychosocial stressors that can shape their professional identity and career development [2]. Interventions promoting PIF can be difficult to employ during clerkships because of the workload and site of clinical duties, assignments, and exam preparation. As a result, we developed Humanities Across Clerkships (HAC), a longitudinal, virtual course during third-year clerkships. Based on constructivism, this course provided space for timely reflection of PIF and career development while minimizing disruptions to clerkships.

We piloted HAC from March 2024 to February 2025 with all third-year medical students (MS3s) at the Penn State College of Medicine (PSCOM) through the Department of Humanities. At PSCOM, medical students start 4–6-week-long clerkship rotations during the third year of medical school. The course featured 26 bi-weekly hour-long virtual sessions in small groups with a faculty facilitator, 2 fourth-year student facilitators, and 7–9 MS3s. All small groups met on the same weeks at a time chosen by each group. Clerkship teams excused MS3s from clinical duties during this hour. All facilitators received training on small-group learning and student mistreatment. The MS3s were assigned based on existing advising cohorts. In all sessions, MS3s reflected upon challenges during clerkships. In select sessions, facilitators used session guides and medical narratives assigned as pre-work to prompt discussion of humanities concepts (ex. professionalism and interpersonal relationships) and career development skills (ex. feedback and specialty choice) relating to the clinical environment. In other sessions, MS3s were invited to share memorable experiences from clerkships or to follow up on previous prompts.

To pass the course, participants were graded on three domains: attendance, participation, and completion of writing assignments. Attendance was required for all sessions, with two excused absences allowed. Participation was evaluated by biannual surveys. The survey is a standardized form for all humanities courses at the institution (not previously published) and asked if the MS3s were prepared, respectful, and professional. Three writing assignments asked MS3s to reflect on PIF and career development at the beginning, middle, and end of the year (e.g., “Describe a memorable patient encounter. What about it was memorable? What ways did it impact what your vision of the way a doctor should practice?”). The assignments were graded based on timely submission and response completion and not on content to preserve the course as a safe space; the facilitators debriefed on them and course objectives in the last session. Facilitators tracked attendance, completed the surveys, and graded the assignments. A remediation assignment was given to any MS3 who did not initially pass the course, with similar questions regarding PIF, career development, and professionalism. Program evaluation comprised an institutional standardized end-of-course feedback form.

In this pilot, 128 MS3s were enrolled and passed the course. Fifty-nine MS3s attended all sessions, but 3 MS3s remediated because of multiple absences. Eighty-nine MS3s completed the program evaluation survey. Fifty-nine MS3s rated the overall quality of the course at least a 4/5, with 5 as excellent. Seventy-three rated its clinical relevance at least a 4/5. Sixty-eight rated small-group sessions at least a 4/5. In narrative comments, students appreciated the course’s reflective, safe space with peers, support of student facilitators, and course flexibility amongst clinical duties with its virtual format and individual group timings. However, areas for improvement included scheduling conflicts with group meeting timings and clerkship didactics and the challenges of bonding amongst group members on a virtual platform. In response, course leadership decided to excuse MS3s from HAC for clerkship didactics; this absence did not count towards the attendance policy. Next year, course leadership plans to have a uniform time for all groups to meet that does not interfere with any clerkship requirement. Next year, groups will meet intermittently in person to foster interpersonal relationships and interactive discussion while keeping other sessions virtual for accessibility. Writing assignments will continue to not be assessed for content.

This innovative longitudinal, virtual course promoted reflection of PIF and career development for MS3s rotating on clerkships. This approach encourages individual and group reflection and efficiently integrates humanities with clinical duties.

## Data Availability

Data are presented in the manuscript. Additional information about the innovation is available upon request.
